# Does Transfusion of Red Blood Cells Impact Germline Genetic Test Results?

**DOI:** 10.3390/jpm10040268

**Published:** 2020-12-09

**Authors:** Maggie A. DiGuardo, Sarah J. Kester, Victor J. Mahaffey, Scott A. Hammel, Katelyn K. Heaser, Christopher D. Hofich, Craig D. Tauscher, Sarah E. Kerr, Jennifer L. Oliveira, Eapen K. Jacob, Ann M. Moyer

**Affiliations:** 1 Division of Transfusion Medicine, Department of Laboratory Medicine and Pathology, Mayo Clinic, Rochester, MN 55905, USA; diguardo.margaret@mayo.edu (M.A.D.); Hammel.Scott@mayo.edu (S.A.H.); Heaser.Katelyn@mayo.edu (K.K.H.); Tauscher.Craig@mayo.edu (C.D.T.); Jacob.Eapen@mayo.edu (E.K.J.); 2 Division of Laboratory Genetics and Genomics, Department of Laboratory Medicine and Pathology, Mayo Clinic, Rochester, MN 55905, USA; Kester.Sarah@mayo.edu (S.J.K.); Hofich.Christopher@mayo.edu (C.D.H.); 3 Division of Hematopathology, Department of Laboratory Medicine and Pathology, Mayo Clinic, Rochester, MN 55905, USA; Mahaffey.Victor@mayo.edu (V.J.M.); oliveira.jennifer@mayo.edu (J.L.O.); 4 Hospital Pathology Associates, Minneapolis, MN 55407, USA; sarah.kerr@allina.com

**Keywords:** transfusion, genetic testing, leukoreduction, transfusion-associated microchimerism, interference

## Abstract

Purpose: molecular testing is often indicated for recently transfused patients. However, there are no guidelines regarding the potential interference from donor DNA or whether it is necessary to wait for a period of time post-transfusion prior to genetic testing. While the majority of patients are transfused in the non-trauma setting using leukoreduced (LR) red blood cell products, the degree of leukoreduction varies among centers and is not universally practiced. Methods: whole blood units collected from anonymous donors were used in an in vitro transfusion model. One unit was split: half being leukoreduced simulating a leukopenic recipient and half left untreated. Donors were simulated by leukoreduced, partially leukoreduced (PLR), or non-leukoreduced units, transfused in 2, 5, or 16 unit equivalents. DNA from the combinations were subjected to short tandem repeat (STR) analysis for chimerism detection. Results: donor DNA was not detectable in any of the LR combinations, but detected in the PLR combinations, ranging from 0.1 to 1.5% donor DNA in the immunocompetent recipient and 6.3–27.8% in the leukopenic recipient. Non-LR donor DNA was also detected (13–95%). Conclusion: donor-derived DNA from leukoreduced blood products is unlikely to interfere with the interpretation of germline genetic testing in immunocompetent recipients but may interfere in immunocompromised recipients.

## 1. Introduction

“My patient just received a transfusion. Is it ok to order a genetic test or do I need to wait?” is a commonly encountered question in the clinical genetics laboratory due to concerns about the possible presence of donor DNA in the transfused blood product(s).

Microchimerism, the presence of two genetically distinct populations within an individual, can occur as a result of natural or iatrogenic processes. Naturally acquired microchimerism arises from twin to twin transfusions in utero or more commonly, as a result of bi-directional cellular exchange during pregnancy between a mother and fetus and is often still present even decades after the pregnancy [1]. Microchimerism can also be induced iatrogenically; typically, the result of a solid organ or hematopoietic stem cell transplants, and less commonly, blood transfusions [1,2,3,4].

Transfusion-associated microchimerism has been well documented in trauma patients receiving both whole and leukoreduced blood products; however, study results have been mixed regarding the presence of donor DNA from the transfusion of leukoreduced (LR) blood components in non-trauma patients [4,5,6,7,8,9,10]. Pre-storage leukoreduction was initially implemented to improve clinical outcome; decreasing febrile reactions, viral transmission, and alloimunization to human leukocyte antigen (HLA) antibodies are all benefits of fewer white blood cells (WBCs). While leukoreduction is designed to remove the white blood cells (WBCs) from a blood donation, the white count after the leukoreduction process is not zero. In fact, the FDA mandates that LR red blood cell (RBC) units only fall below 5 × 10^6^ in 95% of units tested after filtration; and the degree of leukoreduction within the acceptable range can vary among centers depending on the process. A number of filter iterations have been tried in order to achieve these endpoints and the current third and fourth generation pre-storage filtration systems are able to achieve a 3–4 log reduction (99.99%) in the WBC counts, which far surpasses the mandated thresholds set forth by the FDA and The Council of Europe. In addition, while most medical centers currently transfuse leukoreduced red blood cell products for non-trauma patients, it is not a requirement; approximately 70% of RBC and platelet products are pre-storage leukoreduced in the United States [11]. Whole blood transfusions, which are non-leukoreduced (NLR) are also used clinically. 

Molecular genetic testing has drastically increased over the past decade for a wide spectrum of conditions. As of 2017, there were 75,000 commercially available genetic tests with an additional 10 entering the market daily, the majority of which were prenatal with hereditary following closely behind [12]. In addition, a review of the genetic testing claims database shows 1.7 million commercial claims during the three year period 2014–2016. This rapid expansion has been accompanied by significant advances in the technology leading to molecular assays with superb analytic sensitivity and diagnostic accuracy and they are being utilized for diagnostic, therapeutic and prognostic purposes in almost every corner of medicine. Due to the underlying indications for these tests, it is not uncommon for a number of these subspecialty patients to have also received a red blood cell (RBC) transfusion and since the most common specimen collected for molecular testing is peripheral blood, the possibility of microchimerism conveys potential impacts for testing. Some laboratories and providers may delay the testing out of concern for DNA contamination from donor leukocytes while others may not have even considered this potential, particularly if the laboratory is unaware of recent transfusion, and the result interpretation can be confounded or inaccurate in these patients. Furthermore, while buccal swabs are sometimes utilized to avoid potential contamination from donor leukocytes, they are often contaminated by saliva or blood due to improper collection. This presents additional challenges given that the DNA present in saliva or in blood contaminating a traumatic buccal collection is primarily derived from leukocytes, so similar considerations are important for this specimen type as well [13]. Despite the significance of these issues, little information is available to guide providers or laboratories who are considering testing in the setting of transfusion. With that in mind, we sought to determine what quantity, if any, of infused LR, partial-LR (PLR) or non-LR (NLR) RBC units lead to detectable donor DNA in both simulated immunocompetent and compromised patient (recipient) blood samples and how this may impact the spectrum of genetic testing.

## 2. Materials and Methods

### 2.1. Red Blood Cell Unit Collection and Processing

**Figure 1:**
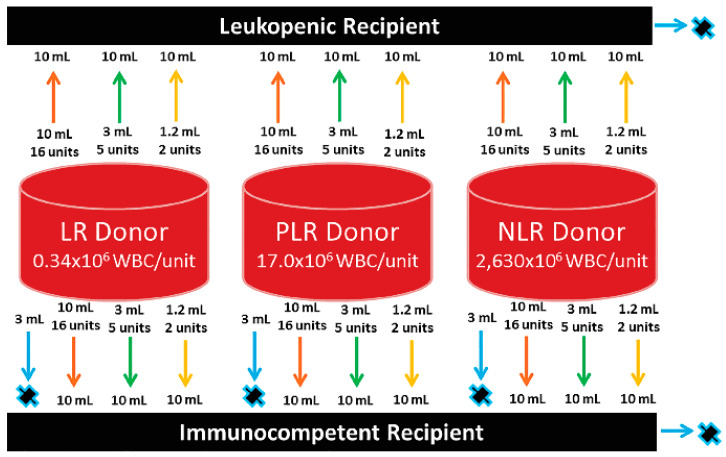
Three whole blood units, representing donors, are shown as red cylinders; the units were processed as leukoreduced (LR), partially leukoreduced (PLR), or nonleukoreduced (NLR), with the residual white blood cell (WBC) counts indicated. A fourth unit was split into 2 aliquots to represent a leukopenic and an immunocompetent recipient (black boxes at top and bottom). Arrows with numbers depict the quantity of donor blood mixed with 10 mL of recipient to simulate transfusion, then chimerism studies were performed. Each “donor” and “recipient” was also tested without mixing (blue arrows with “X”) to identify informative short tandem repeat (STR) markers.

We performed an in vitro spiking study utilizing four whole blood units collected from anonymous donors (Figure 1). All units were collected at the Mayo Clinic Donor Center using the Trima Accel LRS Platelet + Sampler, Plasma, RBC Set (TerumoBCT, Lakewood, CO, USA) and were processed per our institutional standard operating procedure (SOP) for leukoreduction within the Mayo Clinic Blood Components Lab. Two units were leukoreduced at varying levels using a Haemonetics Leukotrap^®^ WB System (Boston, MA, USA) in order to establish both an LR (per our institutional standards) and a PLR unit. The latter was used to simulate the transfusion of units with white counts just outside the AABB criteria for LR, since the degree of LR can vary among institutions, and the content of leukocytes in our institution’s LR units may not reflect that elsewhere. This PLR unit may also mimic an instance of inadequate filtration due to a faulty filter or process given that the AABB standard requires confirmation in only 95% of its’ products and served as another data point from which we could determine when detectable, whether any levels of donor DNA are observed. An NLR unit was used because not all centers provide LR products and NLR whole blood products are also routinely used in the trauma setting. 

WBC counts were measured using the NanoEnTek Adam (Seoul, Korea) per our standard practice. The WBC counts for the LR and PLR units were 0.34 × 10^6^ and 17.0 × 10^6^, respectively. A third unit was processed similarly, but not filtered and was labeled as non-leukoreduced with a WBC count of 2630 × 10^6^. All three units (LR, PLR and NLR) were subsequently treated as our in vitro donors (IVD).

The fourth and final unit collected was divided into equal halves generating two separate but molecularly identical units, which were designated as the in vitro recipients. With the intent of modeling an immunocompromised leukopenic recipient (LR), one of these halves was leukoreduced to the level of 59.32 × 10^6^/unit (corresponding to 0.48 × 10^9^/L in vivo), which is below the threshold of clinical leukopenia (3.4 × 10^9^/L) in vivo. The second half remained un-manipulated and served to represent our immunocompetent normocellular recipient (ICR) (Figure 1).

### 2.2. In Vitro Experiment Calculations and Assumptions

For the in vitro experiments, 10 mL was set to represent the pre-transfusion total blood volume (TBV) for each recipient, assumed to be a 70 kg patient. From this assumption, we then derived the volume of blood in mL that would represent a transfusion of 2, 5, or 16 units. In our model, 1.2 mL of blood equals approximately 2 units and corresponds clinically to an apheresis double RBC product, which is the maximum possible dose of white blood cells from a single donor that could be realistically transfused into a recipient within a 16 week period due to regulations on blood donation. Ten milliliters is equivalent to sixteen units, which is the volume of each of our recipient aliquots. These were intended to represent the combination of blood from two genetically different individuals in a 1:1 ratio, which would clinically model neonatal transfusion situations where one transfused aliquot (from a single donor) could result in a 1:1 ratio as a result of a neonate’s minimal starting TBV. For example, a 3.5 kg infant (average weight for a healthy neonate born in the 50th percentile) would have an approximate TBV of 298 mL; one unit of RBCs can range anywhere from 300 to 550 mL. The five-unit combination was an arbitrary midpoint intended to serve as a point of reference should our initial results necessitate further refinement of our volumes or aliquots. 

The three aliquots, representing 2, 5, and 16 mL, were then removed from each of our 3 IVD units (LR, PLR, and NLR) and combined with a fixed 10 mL aliquot from each of our two recipients for our total of (18) combinations. Donor and recipient samples were mixed and incubated overnight at room temperature, without agitation. An additional aliquot of 3 mL was removed from the three donor units and single recipient unit as well for a baseline DNA signature (Figure 1). Allelic baselines were established for each of the short tandem repeat (STR) chromosomal markers to facilitate chimerism detection from each of the different transfusion scenarios. 

### 2.3. Molecular Testing and Data Analysis

DNA extraction was performed using the QIAgen EZ1 (Hilden, Germany) instrument and chemistry and concentrations were determined using the Promega Quantus Fluorometer (Madison, WI, USA). To assess the degree of detectable donor DNA vs. recipient DNA, chimerism detection was performed by analyzing STR using the GlobalFiler PCR amplification kit (Thermo Fisher Scientific, Waltham, MA, USA) followed by ChimerMarker analysis software (SoftGenetics, State College, PA, USA). Twenty-four STR markers corresponding to donors and/or recipients were evaluated. The area under each signal peak for informative markers (those that differed between the donor and recipient) was used to calculate the percentage donor. The area under the peak corresponding to the donor was divided by the total area of the donor plus recipient peak. This is a clinically validated and commercially available assay thorough Mayo Clinic Laboratories (Test ID: CHIMU) and has a clinically validated lower limit of detection of 5%, but typically detects chimerism (or donor contamination in this study) at levels as low as 1%. This means that it will likely be able to detect donor DNA in concentrations of ≥1% and always >5%. As additional confirmation, we also calculated the expected values for each donor/recipient combination based on the DNA concentrations after extraction and then compared those results to what we observed by STR analysis (Supplemental Table S1). The percentage of donor detected in each of the 18 combinations was then further compared to the sensitivity estimates of various molecular techniques available in the literature to determine the impact of donor DNA contamination on clinical molecular genetic testing.

### 2.4. Comparison to Analytic Sensitivity of Molecular Techniques

A literature search was performed to determine the approximate analytic sensitivity of commonly used molecular techniques. Search terms included the name of each individual technique and “sensitivity” or “analytic sensitivity”. Additionally, this information was obtained by reviewing the validated limits of detection for assays performed at our institution. 

## 3. Results

### In Vitro Transfusion Simulations

In the LR product, 0.013 ng/uL of DNA was detected, whereas 0.836 ng/uL was detected in the PLR product, and 47.0 ng/uL in the NLR product prior to mixing. No donor DNA was detected when any level of transfusion with the LR product was simulated with either the immunocompetent or leukopenic recipients (Table 1 and Table 2). Given that no donor DNA was detectable in the simulated transfusion of any amount of LR product in the leukopenic recipient, the STR chimerism test for the simulation of 16 units of LR product to an immunocompetent recipient, which failed, was not repeated.

When transfusions of PLR product, which had a WBC count above that required to be labeled LR by the AABB, were simulated, donor DNA was undetectable after two units in the immunocompetent recipient. At 5 and 16 units, donor DNA comprised 0.1 and 1.5% of the total DNA, respectively, in the immunocompetent recipient, which is below the limit of detection for most clinical assays evaluating germline genetic variation (Table 1 and Table 3). Transfusions of PLR product to the leukopenic recipient resulted in the detection of 6.3% donor DNA when two units were transfused, 12.2% when five units were transfused, and 27.8% when 16 units were transfused (Table 2 and Table 3). These values would be expected to impact a subset of clinical genetic assays.

The transfusion of two or more units of NLR products resulted in detectable donor DNA in both the immunocompetent and leukopenic recipients. Donor DNA accounted for 13.3% of total DNA when two units were transfused to the immunocompetent recipient, 24.1% when five units were transfused, and 45.1% when 16 units were transfused (Table 1). When the NLR product was transfused to the leukopenic recipient, the percentage of donor DNA ranged from 75.9% (when two units were transfused) to 95.7% (for transfusion of 16 units) (Table 2). Transfusing as little as two units of NLR product to an immunocompetent recipient may result in a level of donor DNA that would be detected by most clinical assays, while levels above two units of PLR, or any quantity of PLR or NLR transfused to a leukopenic recipient, could interfere with results, depending on the platform (Table 3).

## 4. Discussion

This in vitro transfusion model reliably confirmed that donor DNA was not detectable after a simulated transfusion with any volume of the leukoreduced product, while the partially leukoreduced product displayed a very low level range of 0.1–1.5% chimerism in the immunocompetent recipient and a clinically significant (from a testing standpoint) range of 6.3–27.8% in the leukopenic recipient. Donor DNA was observed in each simulated transfusion scenario with the NLR product and would be expected to have a significant impact on testing. Donor-derived DNA from leukoreduced blood products is unlikely to interfere with the interpretation of germline genetic testing in immunocompetent recipients, but depending on the degree and quality of the leukoreduction, may interfere in immunocompromised recipients.

Given the efficiency of pre-storage leukoreduction systems, we did not anticipate that an LR transfusion would lead to significant DNA contamination from the donor, which was supported by the negative results observed in all of the simulations of LR product to both immunocompetent and leukopenic recipient combinations. Previously published experiments have reported similar findings using LR products; a number of these, however, were limited by patient population (critical care patients) and access to only the recipient’s DNA [7,9,18,19]. These results are particularly noteworthy for the leukopenic recipient due to the fact that leukopenic patients comprise a large majority of the patient population receiving therapeutic transfusions and their underlying diseases can necessitate genetic testing.

Despite the fact that no donor DNA was detected in the recipients of LR units, the analysis of the simulated PLR transfusions did reveal low but appreciable levels of donor DNA in the immunocompetent recipient. For the immunocompetent recipient, these results can realistically only be extrapolated to suggest the potential to interfere with germline genetic testing in situations where (1) the recipient is a neonate; (2) the volume transfused is close to a 1:1 ratio with their TBV; and (3) the subsequent genetic test has a limit of detection below 2–3%. While this would not apply to NGS-based hereditary assays, which are commonly indicated in these situations, it may impact the targeted genotyping platforms. These considerations are true as well for the leukopenic recipient of a PLR unit, but from a laboratory perspective, of greater significance was the observation that sequentially increasing levels of donor DNA were detected as the number of units transfused to the leukopenic recipient also increased, starting with just the two-unit aliquot.

The presence of donor DNA could result in either the detection of a variant in the donor, but not present in the recipient (false positive), or could obscure the presence of a variant in the recipient and not present in the donor(s). For example, if the recipient was being tested for Diamond-Blackfan anemia due to pure red cell aplasia, and has a heterozygous *RPS19* variant, a large quantity of donor DNA that is negative for the variant (wild type) could decrease the signal for the variant nucleotide. However, a significant quantity of donor DNA would be required, given that the recipient’s DNA would contain 50% variant and 50% wild-type sequence at that locus; however, if the patient presented with bone marrow failure due to the presence of a heterozygous *FANCA* variant and is leukopenic, a much lower quantity of donor DNA would be required to decrease the variant from the expected 50%. In contrast, if the recipient is a neonate undergoing diagnostic exome sequencing for failure to thrive, and the donor is heterozygous for a *KCNQ1* variant that would predispose to long QT syndrome, the presence of donor DNA may result in a signal; however, again, significant donor DNA would need to be present to reach a level near 50% variant frequency to result in a false positive clinical call. More likely, the laboratory would note the unusual variant frequency and contact the ordering provider for more clinical and family history to understand the unexpected findings and may recommend confirmatory testing with a new blood draw. An adult recipient would only ever receive up to two units from a single donor (a double red blood cell unit), which are often processed using a single filter, due to the regulations surrounding RBC donation. Therefore, for an adult recipient, false positives due to donor variants would be unlikely; however, if the recipient is leukopenic, the 6.3% donor DNA observed could potentially interfere with a sensitive assay. For both immunocompetent and leukopenic recipients, false negatives would be unlikely, unless the patient had low level mosaicism and a massive transfusion. Additionally, as mentioned above, in germline testing, most variants are expected to be present at 50% for heterozygotes or 100% for homozygotes, so the identification of 6.3% donor DNA may not impact results, unless incorrectly attributed to naturally occurring mosaicism.

The focus of this manuscript is germline testing, but similar considerations would apply to somatic testing. Somatic testing is typically directed toward specific variants, which are expected to be present in a healthy blood donor; therefore, false positives would be highly unlikely. If the recipient had a somatic variant, the presence of donor DNA (which again would likely be negative for the somatic variant in a healthy blood donor) may lead to a lower variant allele frequency; however, unless the variant was present at a frequency close to the limit of detection, this would require the presence of a significant amount of donor DNA to have a clinically significant impact.

When applying these results to realistic clinical scenarios, it is important to understand the probability of receiving a PLR unit in practice. As mentioned, AABB standards, per the FDA, mandate that LR blood and blood components shall be prepared by a method known to reduce the leukocyte number to <5 × 10^6^ for RBC and apheresis or pooled platelets and to 8.3 × 10^5^ in whole-blood-derived platelets, demonstrated in >95% of units sampled. The Council of Europe (EC) has an even lower threshold of <1 × 10^6^ leukocytes demonstrated in >90% of WBC-reduced RBC and single-donor platelets. Filtration failures (defined by the inability to meet thresholds of <5 × 10^6^ or <1 × 10^6^ leukocytes) can indeed occur and are attributed to the age of unit, temperature, height of the bag at the time of filtration, filter malfunctions and flow rates [20,21,22,23]. However, Yomtovian et al., utilizing data collected from the 11 sites in the Viral Activation Transfusion Study (VATS), were able to show that only 0.8% (15 units) exceeded 5 × 10^6^ WBCs per unit while approximately 8% exceeded the EC threshold of 1 × 10^6^ [20]. Importantly, 2 out of the 15 were found to have not received any filtration while the remaining 13 were all in the 10^7^ range, indicative of filtration malfunctions; our PLR had a WBC count of 1.7 × 10^7^. Importantly, these numbers fulfilled all quality expectations and were still well within the required passing thresholds of 95 and 90%. Additionally, based on the exceedingly low likelihood of receiving a PLR unit, in practice this possibility should only be considered if contamination is observed in the raw data results and all other causes have been sufficiently ruled out.

As expected, all of the NLR combinations displayed increasing amounts of donor DNA in both recipients. The two-unit transfusion in the immunocompetent recipient had the least amount of donor DNA at 13.3%, a level certainly capable of confounding genetic testing and a clinically realistic scenario since leukoreduction is not a universal practice in the United States and NLR whole blood products are often used in the setting of trauma. The other scenarios are only clinically realistic for a neonate, since, again, an adult patient would not receive greater than two units from the same donor.

This study was limited by the fact that it was an in vitro model and cannot account for the size difference of recipients, immunologic variability, the sequestration of WBCs, laminar flow and turbulence, or the natural physiologic changes that occur with in vivo transfusions. In vivo, an immunocompetent recipient’s immune system would presumably recognize any donor WBCs present in the transfused product and eliminate it. One study using allele-specific PCR assays suggested that 99.9% of donor leukocytes were cleared within the first two days after transfusion. Therefore, most of these factors would not lead to an increase in the quantifiable donor DNA, but rather, a decrease such that the in vitro model presented here represents the highest concentration possible from a transfusion. However, it is important to note that a transient 1-log expansion has been observed on days 3–5 in a previously published study. [24] Therefore, in vivo studies to explore the impact of transfusion on genetic testing over the days after transfusion when this proliferation could occur are warranted. Conversely, immunocompromised recipients may not be able to efficiently mount an immune response to recognize and/or destroy donor WBCs, so there may be a greater chance of engraftment and long-term complications with genetic testing results; however, when clinically indicated, immunocompromised recipients receive irradiated WBCs that cannot proliferate, so our model, which simulates the impact of donor WBCs shortly after transfusion is likely still applicable [25]. Furthermore, our institution is able to leukoreduce, on average, to a level of 3 × 10^5^, which may be different from that of other institutions. That being said, Terumo publishes an average residual leukocyte count of 2 × 10^5^ for its BCT Immugard III-RC filter for whole blood and red cells, so our institutional average seems to be on par with the industry standard. Despite these limitations, similar results have been seen when analyzing the impacts of transfusion on the HLA typing of deceased donors, further confirming our results. [8,10]

With some exceptions, our in vitro results suggest that STR analysis with a limit of quantification of 1% is sufficient to be reliably applied as a surrogate marker of contamination risk for the majority of commercially available molecular assays and the following recommendations can be applied when triaging these situations both pre and post analytically:

1. Leukoreduced blood products are exceedingly unlikely to affect genetic testing in patients of any age. Information regarding this can be easily obtained from the institution’s blood bank or laboratory director;
2. Transfusion of non-leukoreduced products can lead to appreciable amounts of donor DNA and these situations need further investigation including information such as the number of products transfused, the length of time since the last transfusion, and the clinical scenario.

In conclusion, the standard transfusion of two leukoreduced RBC units to an adult immunocompetent or immunocompromised recipient is unlikely to impact genetic testing results; however, in other scenarios (e.g., neonates, trauma patients receiving non-leukoreduced whole blood) there is a potential for donor DNA to impact the genetic testing results. Consultation with the genetic testing laboratory and the blood bank, along with clinical correlation if testing is performed, is recommended.

## Figures and Tables

**Table 1:** Percentage of donor DNA detected in the simulated transfusions of LR, PLR, and NLR red blood cells (RBCs) to an immunocompetent recipient. The amount of donor blood mixed with 10 mL of recipient is listed in the first column, while the type of donor is indicated in the top row of each column.

Immunocompetent Recipient			
DONOR	LR	PLR	NLR
1.2 mL (2 units)	0.0%	0.0%	13.3%
3.0 mL (5 units)	0.0%	0.1%	24.1%
10 mL (16 units)	No Amp	1.5%	45.1%

**Table 2:** Percentage of donor DNA detected in the simulated transfusions of LR, PLR, and NLR RBCs to a leukopenic recipient. The amount of donor blood mixed with 10 mL of recipient is listed in the first column, while the type of donor is indicated in the top row of each column.

Leukopenic Recipient			
DONOR	LR	PLR	NLR
1.2 mL (2 units)	0.0%	6.3%	75.9%
3.0 mL (5 units)	0.0%	12.2%	88.1%
10 mL (16 units)	0.0%	27.8%	95.7%

**Table 3:** Approximate analytic sensitivity of commonly used molecular techniques.

Technique	Approximate Analytic Sensitivity
Sanger Sequencing	10–20% [14]
Restriction Fragment Length Polymorphism Analysis	0.1–1% [15]
Pyrosequencing	5% [14]
Next Generation Sequencing (germline)	5–10% *
Next Generation Sequencing (somatic)	5% **
Real-Time PCR with Melt Curve Analysis	1–10% [14]
Single-Stranded Conformational Polymorphism Analysis with Denaturing High Performance Liquid Chromatography	1–5% [16]
Microsatellite Short Tandem Repeat Analysis by Capillary Electrophoresis	1% ***
Amplification Refractory Mutation System Analysis	0.05–0.1% [16]
Droplet Digital PCR	0.25% [17]

* varies based on read depth and pipeline. ** varies based on read depth and pipeline; somatic variant filtering tends to be more permissive. *** institutionally validated.

## Supplementary Materials

The following are available online at https://www.mdpi.com/2075-4426/10/4/268/s1, Table S1: Expected and Observed Chimerism After Simulated Transfusion.
